# Heterogeneity of the Alpha-Smooth Muscle Actin Tumor Score in Breast Cancer Cells Significantly Affects Tumor Invasiveness, Recurrence, and Patient Survival

**DOI:** 10.7759/cureus.75908

**Published:** 2024-12-17

**Authors:** Mihaela-Maria Pasca Fenesan, Andrei Alexandru Cosma, Eugen Melnic, Anca Maria Cimpean, Gabriel Veniamin Cozma, Alina Gabriela Negru

**Affiliations:** 1 Department of Microscopic Morphology/Histology, Doctoral School in Medicine, Department of Clinical Oncology, Victor Babeș University of Medicine and Pharmacy, Timisoara, ROU; 2 Department of Pathology, Nicolae Testemitanu State University of Medicine and Pharmacy, Chișinău, MDA; 3 Department of Microscopic Morphology/Histology, Center of Expertise for Rare Vascular Disease in Children, Emergency Hospital for Children Louis, Center of Genomic Medicine, Research Center for Pharmaco-Toxicological Evaluation, Victor Babeș University of Medicine and Pharmacy, Timisoara, ROU; 4 Discipline of Surgical Semiology I and Thoracic Surgery, Department of Surgery I, Thoracic Surgery Research Center, Victor Babeș University of Medicine and Pharmacy, Timisoara, ROU; 5 Department of Cardiology, Victor Babeș University of Medicine and Pharmacy, Timisoara, ROU

**Keywords:** alpha smooth muscle actin, breast cancer, invasion, molecular subtypes, recurrence, survival

## Abstract

Background: Alpha-smooth muscle actin (αSMA) has been widely investigated in malignancies, primarily concerning its expression in cancer-associated fibroblasts (CAFs) inside the tumor stroma. Microscopic examination indicates that αSMA expression is not confined to the tumor stromal compartment but is also present in a subset of tumor cells, and this expression correlates with an enhanced invasive phenotype of malignant cells from lung, liver, or ovarian malignancies. Information on actin expression in breast cancer (BC) cells is scarce, and its influence on clinicopathological characteristics remains ambiguous due to conflicting findings in the literature.

Objective: To examine the αSMA tumor score in breast cancer cells utilizing digital image analysis (DIA) methodologies and to critically analyze the varying effects of αSMA tumor score values on clinicopathologic parameters, particularly focusing on tumor cell invasiveness, recurrence, and survival.

Materials and methods: Double immunostaining for CD34 and αSMA was conducted on 53 breast cancer cases that were thoroughly characterized in relation to clinicopathologic data. Double immunostaining for CD34 and αSMA demonstrated different distribution patterns of both markers in normal and breast cancer tissues. DIA data about αSMA tumor cell density, intensity, tumor score, and histological score were correlated with clinicopathological factors.

Results: We delineated three unique breast cancer subgroups based on αSMA tumor scores: a 9.43% low-expressing subgroup (αSMA_TS^low^, score 4), a 35.07% medium-expressing subgroup (αSMA_TS^med^, scores 5 and 6), and a 55.5% high-expressing subgroup (αSMA_TS^high^, scores 7 and 8). Stromal immature vessels and tertiary lymphoid structures (TLS) exhibited a strong correlation with αSMA_TS^low^, whereas recurrence, perineural, and lymphovascular invasion strongly influenced the αSMA_TS^med^ and αSMA_TS^high^ subgroups. The αSMA_TS^med^ subgroup demonstrated the most heterogeneity with the influence of αSMA-expressing breast cancer cells on tumor size, nodal status, perineural and lymphovascular invasion, menopausal status, recurrence, and survival. Most of the cases from the αSMA_TS^low ^subgroup had Luminal B and Luminal B-HER2 phenotypes, while triple-negative breast cancer (TNBC) represented one-third of all cases in the αSMA_TS^high ^subgroup.

Conclusion: αSMA-expressing breast cancer cells variably affect malignant growth, invasion, and recurrence, highly contingent upon their density and expression intensity. The current investigation identified an αSMA_TS^med^ BC subgroup that appears to promote invasiveness, recurrence, and survival in breast cancer. Our data indicate that αSMA BC-expressing cells play a dual role in BC progression, contingent upon their percentage and expression intensity; however, further research is required to elucidate the factors and mechanisms responsible for their accumulation and/or transdifferentiation in malignant breast tissue.

## Introduction

Breast cancer represents the most prevalent form of malignancy among women globally and exhibits a curable rate of approximately 70-80% in patients diagnosed with early-stage, non-metastatic disease. The presence of advanced breast cancer accompanied by distant organ metastases is regarded as incurable with the therapies that are presently accessible. At the molecular level, breast cancer presents as a heterogeneous disease characterized by various molecular features, including the activation of the human epidermal growth factor receptor 2 (HER2, encoded by ERBB2), the activation of hormone receptors (such as the estrogen receptor and progesterone receptor), and/or mutations in the BRCA genes [1].

Breast cancer cells have a high ability to migrate, invade the local surrounding tissues and lymph nodes, and initiate distant metastases. Cell movement is based on the actin cytoskeleton rearrangement induced by several local and general factors [2, 3].

Actin is a fundamental structural protein constituting the cytoskeleton of cells, and it is involved in processes such as division, migration, and vesicle trafficking. It consists of six distinct isoforms specific to various cell types: ACTA1, ACTA2, ACTB, ACTC1, ACTG1, and ACTG2. Altered expression of actin isoforms has been shown in numerous malignancies, prompting us to propose that it could function as an early diagnostic marker for cancer [4]. Alpha-smooth muscle actin (αSMA, ACTA2) plays a role in the generation of mechanical tension by cells and the preservation of cellular morphology. αSMA is crucial in the migration and invasion of malignant cells [5]. Elevated αSMA expression facilitates the epithelial-mesenchymal transition (EMT) associated with tumor progression and metastasis in lung and pancreatic malignancies [6, 7].

In normal human breast tissue, αSMA expression is restricted to the myoepithelial cells surrounding secretory components and ductal structures [8]. Myoepithelial cells serve as a dynamic barrier to the dissemination of epithelial cells [8, 9].

αSMA expression has been intensely studied in breast cancer stromal-associated fibroblasts (CAFs) [10, 11], and it was proven that αSMA-positive CAFs increase breast cancer tumor stroma and influence tumor progression, metastatic potential, prognosis, and patients' overall survival [12-14]. Recent data also stated that αSMA-positive circulating CAFs have the ability to travel through the bloodstream, significantly impacting breast cancer metastatic potential and influencing tumor prognosis and overall survival [15].

By contrast to the extensive research on αSMA in the breast cancer stromal compartment, its presence and role in breast cancer cells are far less studied. Most related data, derived from experimental research, describe the interrelation between αSMA-expressing breast cancer cells and other factors involved in BC initiation, progression, invasion, and metastasis. Kim et al. demonstrated that TP53 enhances the expression of αSMA in breast cancer cells that exhibit resistance to tamoxifen [16]. Jeon et al. demonstrated that the induction of ACTA2 through the dimerization of EGFR and HER2 is regulated by a JAK2/STAT1 signaling pathway and that abnormal expression of ACTA2 increases the invasiveness and metastasis of breast cancer cells [17]. In a study published by Yadav et al., the authors reported an atypical Luminal A BC case overexpressing αSMA in tumor cells, showing a poor prognosis compared to other similar cases tested for the same parameters [18]. The capacity for self-renewal and the ability to form spheres from breast cancer stem-like cells originating from human primary invasive ductal carcinoma were associated with high αSMA expression in BC cells [19]. lncRNA ACTA2-AS1 serves as a prognostic indicator of malignancy and adverse outcomes in triple-negative breast cancer, influencing tumor progression through the modulation of miR-532-5p [20].

Despite the indirect evidence of αSMA’s role in BC cell behavior, no well-defined data related to its impact on clinicopathologic parameters have been found in the literature. During one of our previous studies related to the immunohistochemical expression of αSMA in the BC stromal compartment, we observed that malignant areas also contain αSMA-positive malignant cells showing high variability in their density and intensity of expression. We wanted to determine whether this heterogeneous expression may have any impact on clinicopathologic parameters. Based on previously described evidence, we initiated the present research to assess αSMA BC cell heterogeneity using immunohistochemistry (IHC) and Digital Image Analysis (DIA) techniques. αSMA tumor score values were then critically analyzed and correlated with clinicopathologic parameters (age, IMC, TNM staging, perineural and lymphovascular invasion, stromal components, molecular subtypes, survival, recurrence, and menopausal status), particularly focusing on tumor cell invasiveness, recurrence, and survival.

## Materials and methods

Criteria for patient selection and corresponding ethical implications

This study is part of a larger retrospective analysis involving 150 formalin-fixed paraffin-embedded (FFPE) breast cancer specimens obtained from women diagnosed with ductal invasive carcinoma via histopathological assessment, identified between 2015 and 2022, and aged 32 to 85 years. Two separate pathologists performed a follow-up study of the FFPE samples to confirm the diagnosis and identify cases appropriate for IHC. A molecular profile was created for each case using immunohistochemical markers. The markers included the estrogen receptor (ER), the progesterone receptor (PR), the Proliferation Index (Ki67), and an evaluation of the human epidermal growth factor receptor 2 (HER2). This was crucial for clarifying the different molecular subtypes of breast cancer. To achieve the goals of this inquiry, we selected 53 individuals who displayed a thorough clinical, histological, and therapeutic profile considered beneficial for our analysis. This study examines age, menopausal status, breast cancer molecular subtype, tumor grade (G), Nottingham Prognostic Index (NPI), lymphovascular invasion, perineural invasion, recurrence, and TNM staging, all of which are relevant clinicopathologic and therapeutic factors. We identified αSMA-positive (αSMA+) breast cancer cells inside malignant tumor regions and classified stromal tumor blood vessels as either immature (CD34+/αSMA-) or mature (CD34+/αSMA+) to ascertain connections with the density and distribution of αSMA+ tumor cells. At the time of diagnosis, imaging modalities verified the absence of any verifiable history of metastases in the patients. Informed consent was obtained from each patient using a standardized form that had been evaluated and approved by the Research Ethics Council of the Victor Babeș University of Medicine and Pharmacy in Timișoara (No. 49/28.09.2018).

Initial processing, histological assessment, and criteria for the selection of FFPE specimens for IHC evaluation

Initially, tumor samples were obtained for diagnostic purposes before the initiation of treatment, which included a mastectomy and needle core biopsy. A sample of the tumor, including the surrounding tissue that represented the whole, was chosen for examination. After acquiring breast cancer tissue samples, the specimens were stored in buffered formalin for 24 to 48 hours, thereafter embedded in paraffin using the standard process. The FFPE block was precisely sectioned to a thickness of three micrometers, and the resulting sections were carefully mounted onto glass slides. A slide was procured from each case for histopathologic investigation and stained with hematoxylin and eosin. Two separate pathologists evaluated the hematoxylin and eosin-stained slides pertinent to the case to validate the initial histopathological diagnosis and appraise the tissue quality. This was essential for ensuring the appropriate selection of patients for IHC. Vimentin (clone V9) immunostaining was utilized to evaluate the overall quality of the tissue. Cases demonstrating positive vimentin staining in the tumor stroma were considered suitable for selection and subsequent immunohistochemical analysis.

Immunohistochemistry

IHC was conducted on three-micrometer-thick sections with an autostainer manufactured by Leica Biosystems, based in Newcastle upon Tyne, United Kingdom. The Novocastra Bond Epitope Retrieval Solutions 1 and 2 were utilized in the unmasking method (Leica Biosystems, Newcastle Ltd, Newcastle upon Tyne NE12 8EW, UK). A 3% hydrogen peroxide solution was used to inhibit endogenous peroxidase activity for five minutes. A twofold immunostaining approach was applied to multiple tissue specimens to examine the presence and location of the αSMA+ response in breast cancer cells. A comprehensive analysis of the unique features of mature and immature tumor blood vessels originating from the breast cancer stromal component was performed and previously evaluated. CD34 mouse anti-human monoclonal antibodies (clone QBEnd 10, Leica Biosystems, Newcastle upon Tyne, UK) were applied for 30 minutes at ambient temperature to target the endothelium of tumor vasculature. Additionally, we used αSMA mouse anti-human monoclonal antibodies (clone 1A4, Leica Biosystems, Newcastle upon Tyne, UK), with a 30-minute incubation at room temperature. The implementation of the IHC approach required the use of visualization systems, namely the Bond Polymer Refine Detection System DAB and the Bond Polymer Refine Red Detection System. For CD34, the vessel endothelium was considered an internal positive control. For SMA, perivascular cells surrounding stromal capillaries were considered positive controls. Negative control was obtained by omitting the incubation with the primary antibody. For additional DIA, we focused on the cytoplasmic expression of αSMA in tumor cells and the concurrent expression of CD34/αSMA in immature versus mature stromal blood vessels proximal to tumor regions.

Image acquisition and DIA

**Figure 1:**
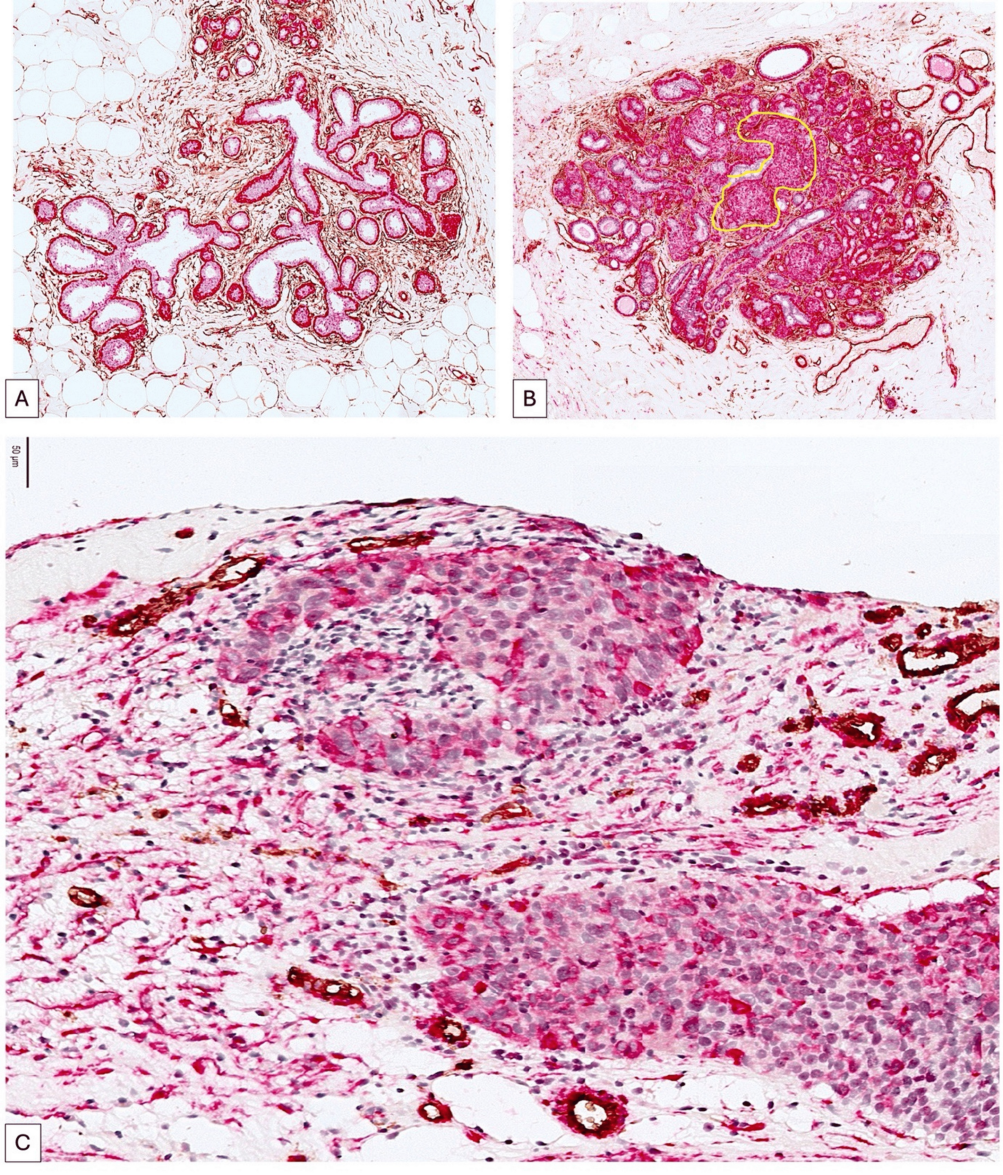
αSMA expression in the normal TDLU (A), early stages of malignant transformation (B, ×100 magnification), and in the tumor cells from invasive carcinoma (C). Note that in normal TDLU, actin expression is restricted to the continuous myoepithelial cell layer surrounding the secretory and ductal components (A, SMA, red, ×100 magnification). Variability in the intensity and density of αSMA-positive tumor cells can be observed in Figure C (×400 magnification). αSMA: alpha-smooth muscle actin, TDLU: terminal duct lobular unit, SMA: smooth muscle actin.

The IHC samples were scanned using a Grundium OCUS 20 Microscope (Grundium, Tampere, Finland) and subsequently stored in the Case Center Slide Library as SVS files (3DHistech, Budapest, Hungary). A project was initiated by importing all slides stained with CD34/SMA and CD34 alone into QuPath version 0.4.3, an open-source platform intended for the bioimage analysis of microscopic slides. The slides were evaluated using integrated software and its supplemental capabilities, including Fiji and Vascular Analysis, to enable an accurate evaluation of tumor stromal blood vessels. In summary, we defined 3-5 regions of interest (ROIs) (Figure 1A) inside the malignant areas using the brush tool, which facilitated the most accurate delineation of tumor regions where αSMA tumor cells were discovered. The DIA analysis began with a pre-processing phase that included the determination of stain vectors (Figure 1B). The next stage of the analysis entailed identifying cells by selecting the positive cell detection option and configuring cell and intensity parameters. The detection image was set up to measure the sum of optical density with a designated pixel size of 0.5 μm and a cell expansion of 1.988 μm, excluding the nucleus. The intensity threshold parameters comprised a score compartment and three evaluative levels: weak (+1, shown in yellow), moderate (+2, marked in orange), and high (+3, denoted in red). All cells highlighted in blue were classified as negative (Figure 1C, D). During automated scoring, the QuPath program provided a cell count, as well as the percentage of both αSMA-positive and αSMA-negative breast cancer cells. It offered distinct density and intensity scores, along with a consolidated Stromal Score (SS), similar to the Allred score, which amalgamates the intensity and density of positively detected cells. Additionally, the conclusive assessment performed using QuPath analysis included a histological score (H-score). In this study, we utilized the intensity of positive αSMA breast cancer cells, their density, and the Allred Score to attain a more accurate evaluation and association with clinicopathologic criteria.

Analytical assessment of statistical data

The statistical analysis was performed using JAMOVI software (The jamovi Project, Sydney, Australia) on macOS systems. The findings obtained from DIA were associated with genetic subtypes of breast cancer, menopausal state, immature and mature stromal blood vessels, tertiary lymphoid structures previously evaluated by us, recurrence, lymphovascular and perineural invasion, and age. A statistical correlation was assessed and considered significant for a p-value of 0.05 or lower.

## Results

Comprehensive analysis of αSMA expression in normal and malignant breast tissues

The dual immunostaining for CD34 and αSMA demonstrated a distinct expression of both markers in normal and malignant breast cancer tissues. The normal terminal ductal lobular unit (TDLU) expresses αSMA in the continuous layer of myoepithelial cells that delineate both secretory and ductal components (Figure 1A). The intralobular and interlobular stroma exhibit elevated CD34 expression, primarily linked to normal stromal fibroblasts. The accumulation of αSMA+ cells within the TDLU, as illustrated in Figure 1B, often indicates the onset of benign breast tissue transformation; however, the discontinuity of the αSMA+ myoepithelial layer serves as a microscopic indicator of breast cancer invasion. Upon the onset of invasive breast cancer, αSMA typically diminishes in tumor cells while concurrently elevating in the stromal compartment, specifically in a subtype of cancer-associated fibroblasts (Figure 1C).

Within the malignant regions of BC, we noted a varied fraction of αSMA+ cells characterized by heterogeneous distribution and expression intensity. We anticipated that the heterogeneity of expression and localization of these αSMA+ tumor cells may influence other tumor characteristics with potential prognostic significance (Figure 1C).

We evaluated intratumoral αSMA expression utilizing the QuPath platform, a digital image analysis software application that enables precise data acquisition on the percentage of αSMA positive cells, as well as the integration of expression intensity and density into a final αSMA tumor score (αSMA_TS).

**Figure 2:**
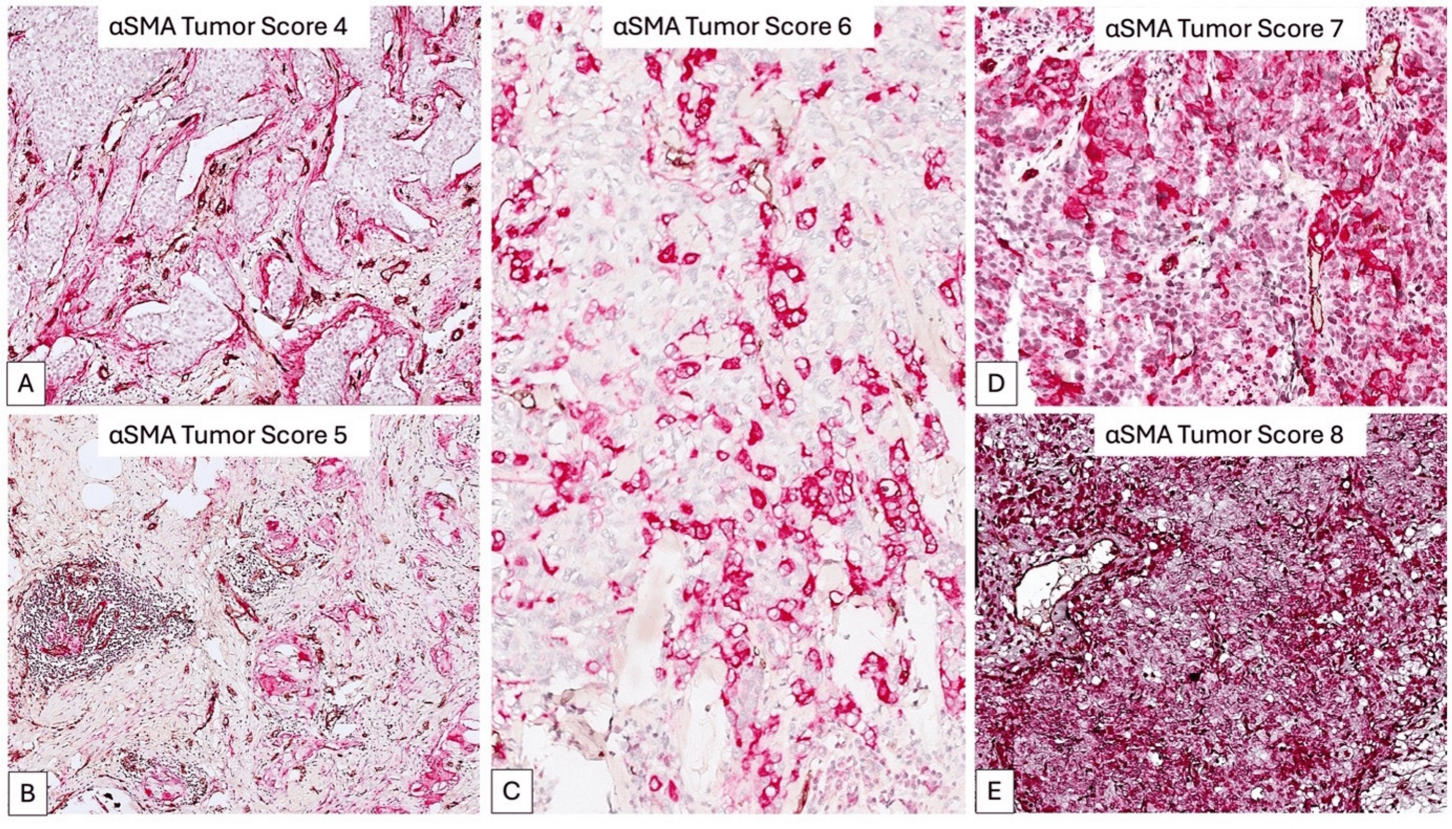
αSMA tumor score (SMA_TS) according to the density and intensity of αSMA expression in tumor cells. Digital Image Analysis defined five scores of αSMA expression, ranging from the lowest score (A), to medium scores (B, C), and high scores (D, E). Based on these scores, we stratified our cases for further analysis. αSMA: alpha-smooth muscle actin.

Utilizing the α SMA_TS value, we delineated three unique breast cancer subgroups: low expressing subgroup (αSMA_TS^low^, 9.43% of all cases with a score of 4, Figure 2A), medium expressing subgroup (αSMA_TS^med^, 35.07% of cases with scores of 5 and 6, Figure 2B,C), and high expressing subgroup (αSMA_TS^high^, 55.5% of cases with scores of 7 and 8, Figure 2D,E) (Table 1).

**Table 1:** The percentage of cases distribution according to the three subgroups. Note that more than half of the cases had a high expression of SMA. αSMA_TS^low^: low αSMA expression subgroup, scored as 4; αSMA_TS^med^: medium αSMA expression subgroup scored as 5 and 6; αSMA_TS^high^: high αSMA expression subgroup.

Subgroup	αSMA_TS^low^	αSMA_TS^med^	αSMA_TS^high^
Tumor score	4	5-6	7-8
% Cases	9.43%	35.07%	55.5%

The impact of αSMA_TS on clinicopathologic parameters

For each subgroup, we analyzed the variability of αSMA_TS interrelation with stromal components (mature/immature blood vessels and tertiary lymphoid structures-TLS) but also with the following clinicopathologic parameters: age, IMC, weight, menopausal status, survival, TNM staging parameters, presence of lymphovascular and perineural invasion, BC molecular subtypes, tumor grade, Nottingham score, HER2 status, and recurrence. None of the cases had distant metastases at the time of initial diagnosis.

For the αSMA_TS^low^ subgroup, all cases were of Luminal B, HER2, and Luminal B-HER2 subtypes. We did not detect any Luminal A or TNBC cases. No perineural (PNI) or lymphovascular (LVI) invasion was detected in any case, nor were nearby nodal (N) metastases. TLS presence and a high number of immature stromal blood vessels (IBV, CD34+/SMA-) were significantly correlated with a low percentage of αSMA-positive tumor cells (Table 2).

**Table 2:** Correlation matrix in between percentage of SMA expressing cells and H-score with lymphoid (TLS) and vascular (IBV) components of the BC stromal compartments. *Significant correlation. **Strongly significant correlation. %SMA+T.Cells: percentage of positive SMA tumor cells in the selected area; H-Score: histological score; TLS: tertiary lymphoid structures; IBV: immature blood vessels with no SMA coverage. Note: *p < .05, **p < .01, ***p < .001, one-tailed.

Components	Variables	%SMA+T.Cells	H-Score
TLS	Pearson's r	0.963**	0.466
	p-value	0.004	0.214
	95% CI upper	1.000	1.000
	95% CI lower	0.679	-0.577
	Spearman's rho	0.866*	0.866*
	p-value	0.029	0.029
	Kendall's Tau B	0.775*	0.775*
	p-value	0.042	0.042
IBV	Pearson's r	0.865	-0.056
	p-value	0.029	0.536
	95% CI upper	1.000	1.000
	95% CI lower	0.147	-0.839
	Spearman's rho	0.975	0.410
	p-value	0.002	0.246
	Kendall's Tau B	0.949	0.316
	p-value	0.011	0.224

**Figure 3:**
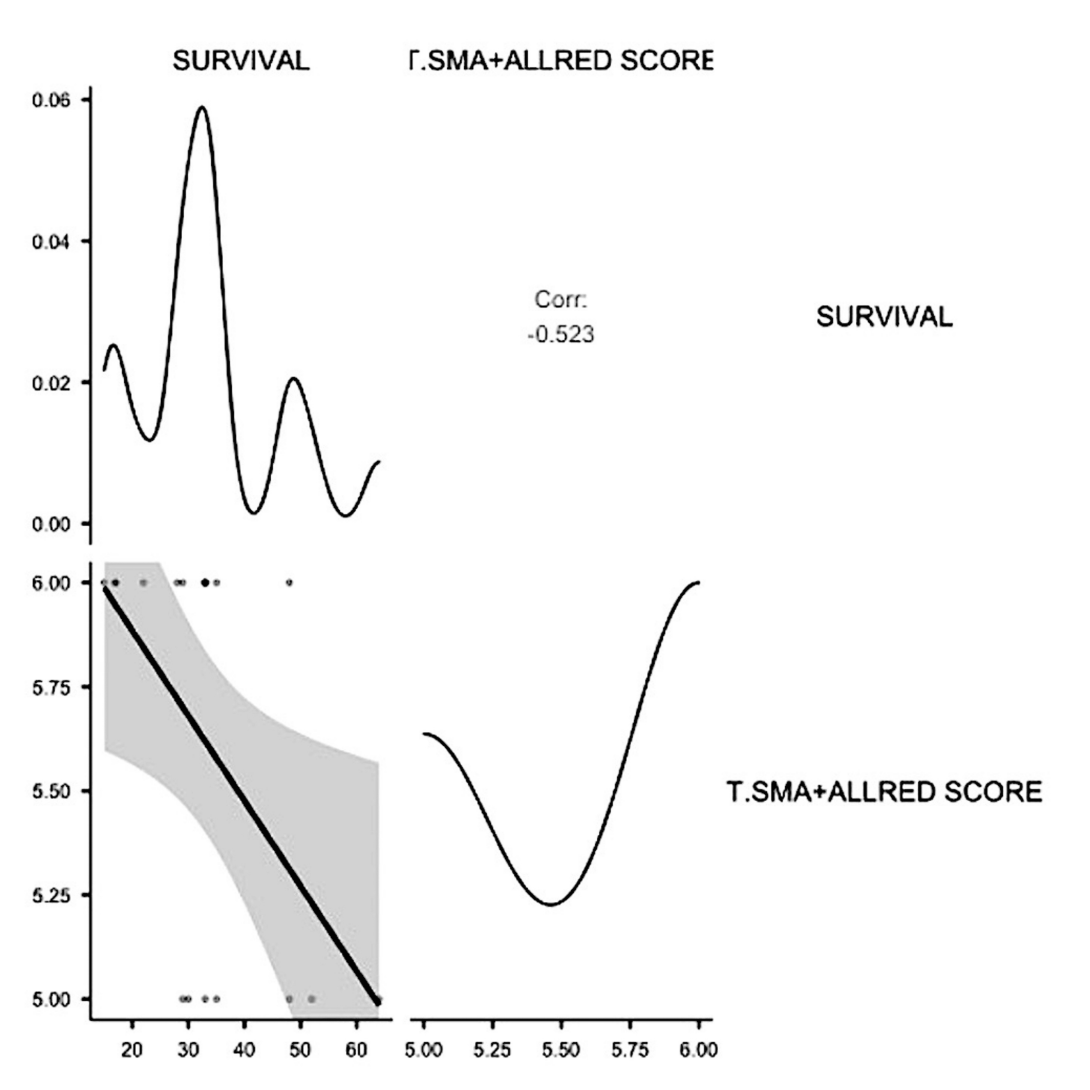
Correlation plot of SMA tumor score and survival. SMA: smooth muscle actin.

No other significant correlations were detected for the αSMA_TS^low^ subgroup. The αSMA_TS^med^ subgroup included 22.22% Luminal A cases, 38.88% Luminal B cases, 11.11% Luminal B-HER2 cases, and 27.77% TNBC cases. No HER2-type cases were reported for this subgroup. Tumor size (T) was significantly correlated with the intensity of αSMA expression in tumor cells (p = 0.005) but not with αSMA_TS. Nodal metastases highly increased in the αSMA_TS^med^ subgroup with a score of 6. For this group, a significant correlation between N, the percentage of αSMA-positive cells (p = 0.048), and αSMA_TS (p = 0.05) was observed. However, the most relevant findings for this subgroup were the significant impact of αSMA-positive tumor cell presence on LVI, PNI, recurrence (R), and survival. Most of the cases with LVI, PNI, and recurrence had a score of 6. A total of 85.74% of cases with a score of 5 had a survival rate ranging between 30 to 64 months, compared with 36.36% of cases scored as 6, where the survival rate ranged between 30 to a maximum of 48 months. Thus, an inverse correlation between survival and αSMA-positive tumor cells was reported in the present study (Table 3 and Figure 3).

**Table 3:** Correlation matrix between tumor score and survival. An inverse significant correlation has been observed. α SMA_TS: tumor score for SMA-positive tumor cells. Note: *p < .05, **p < .01, ***p < .001, one-tailed.

	Variables	Survival
αSMA_TS	Pearson's r	-0.523*
	p-value	0.013
	95% CI upper	-0.155
	95% CI lower	-1.000
	Spearman's rho	-0.498*
	p-value	0.018
	Kendall's Tau B	-0.429*
	p-value	0.020

Strong positive significant correlations have been registered for the αSMA_TS^med^ subgroup related to LVI, PNI, and R (Table 4).

**Table 4:** Correlation matrix for αSMA_TS medium-expressing subgroup. Strong and powerful correlations have been found between LVI, PNI, and R with parameters of SMA expression assessed by the Digital Image Analysis software QuPath. LVI: lymphovascular invasion; PNI: perineurial invasion; R: recurrence; %SMA+T.Cells: percentage of SMA positive tumor cells reported to the total number of cells; H-Score: histological score; SMA.D: density of SMA positive tumor cells; SMA.I: intensity of SMA positive tumor cells; αSMA_TS: tumor score for SMA positive tumor cells. Note: *p < .05, **p < .01, ***p < .001, one-tailed.

		LVI	PNI	R
%SMA+T.Cells	Pearson's r	0.623**	0.685***	0.355
	p-value	0.003	<0.001	0.074
	95% CI upper	1.000	1.000	1.000
	95% CI lower	0.295	0.391	-0.053
	Spearman's rho	0.696***	0.772***	0.417*
	p-value	<0.001	<0.001	0.043
	Kendall's Tau B	0.584**	0.648***	0.349*
	p-value	0.002	<0.001	0.043
H-Score	Pearson's r	0.610**	0.542*	0.408*
	p-value	0.004	0.010	0.046
	95% CI upper	1.000	1.000	1.000
	95% CI lower	0.277	0.180	0.009
	Spearman's rho	0.482*	0.500*	0.388
	p-value	0.021	0.017	0.056
	Kendall's Tau B	0.404*	0.419*	0.325
	p-value	0.023	0.020	0.055
SMA.D.	Pearson's r	0.659**	0.732***	0.463*
	p-value	0.001	<0.001	0.027
	95% CI upper	1.000	1.000	1.000
	95% CI lower	0.350	0.468	0.076
	Spearman's rho	0.675**	0.767***	0.485*
	p-value	0.001	<0.001	0.021
	Kendall's Tau B	0.660**	0.750***	0.474*
	p-value	0.003	<0.001	0.023
SMA.I	Pearson's r	0.000	-0.378	-0.239
	p-value	0.500	0.939	0.830
	95% CI upper	1.000	1.000	1.000
	95% CI lower	-0.401	-0.676	-0.584
	Spearman's rho	0.000	-0.378	-0.239
	p-value	0.500	0.939	0.830
	Kendall's Tau B	0.000	-0.378	-0.239
	p-value	0.500	0.940	0.838
αSMA_TS	Pearson's r	0.798***	0.564**	0.357
	p-value	<0.001	0.007	0.073
	95% CI upper	1.000	1.000	1.000
	95% CI lower	0.583	0.211	-0.051
	Spearman's rho	0.798***	0.564**	0.357
	p-value	<0.001	0.007	0.073
	Kendall's Tau B	0.798***	0.564*	0.357
	p-value		0.010	0.071

The αSMA_TS^high^ subgroup includes more than half of our total number of αSMA-positive BC cases. For all cases in this subgroup, we observed the highest density of αSMA-positive tumor cells, noted with a score of 5. Tumor cell αSMA expression intensity was variable among cases with αSMA_TS scores of 7 and 8. A total of 16.66% of cases had a Luminal A molecular phenotype, 33.33% Luminal B, 10% Luminal B-HER2, 6.66% HER2, and 33.34% TNBC subtype.

Our data showed that cases in the αSMA_TS^high^ subgroup with strong actin expression in tumor cells significantly overlapped with menopausal status (Table 5).

**Table 5:** Correlation matrix between menopausal status and tumor score for high SMA expressing subgroup. αSMA_TS: tumor score for SMA-expressing cells. Note: *p < .05, **p < .01, ***p < .001, one-tailed.

		Menopausal status
αSMA_TS	Pearson's r	0.342*
	p-value	0.032
	95% CI upper	1.000
	95% CI lower	0.040
	Spearman's rho	0.342*
	p-value	0.032
	Kendall's Tau B	0.342*
	p-value	0.033

For the αSMA_TS^high^ subgroup scored 7, several inverse correlations were reported related to tumor size (T2-T4), proliferative status (Ki67), LVI, PNI, and R (Table 6).

**Table 6:** Correlation matrix of tumor parameters for high expressing subgroup. %SMA+T.Cells: the percentage of SMA-positive tumor cells; H-Score: histological score; T: tumor size; Ki67: proliferation index; LVI: lymphovascular invasion; PNI: perineural invasion; R: recurrence. Note: *p < .05, **p < .01, ***p < .001, one-tailed.

		T	Ki67	LVI	PNI	R
%SMA+T.Cells	Pearson's r	-0.717**	-0.485*	-0.509*	-0.826***	-0.758***
	p-value	0.002	0.039	0.032	<0.001	<0.001
	95% CI upper	-0.384	-0.034	-0.065	-0.591	-0.459
	95% CI lower	-1.000	-1.000	-1.000	-1.000	-1.000
	Spearman's rho	-0.433	-0.396	-0.431	-0.608*	-0.669**
	p-value	0.061	0.080	0.062	0.011	0.004
	Kendall's Tau B	-0.325	-0.287	-0.365	-0.514*	-0.566**
	p-value	0.069	0.077	0.060	0.014	0.008
H-Score	Pearson's r	-0.578*	-0.471*	-0.327	-0.795***	-0.623**
	p-value	0.015	0.045	0.127	<0.001	0.009
	95% CI upper	-0.162	-0.016	0.156	-0.528	-0.230
	95% CI lower	-1.000	-1.000	-1.000	-1.000	-1.000
	Spearman's rho	-0.362	-0.517*	-0.196	-0.608*	-0.453
	p-value	0.102	0.029	0.251	0.011	0.052
	Kendall's Tau B	-0.273	-0.354*	-0.166	-0.514*	-0.383
	p-value	0.106	0.040	0.240	0.014	0.051

A direct, positive, significant correlation was observed between the molecular subtype (TNBC) and the αSMA_TS^high^ subgroup, scored as 7 for partial %SMA+ T.Cells and showing a strong H-score (Table 7).

**Table 7:** Correlation matrix between the TNBC molecular subtype with SMA-expressing cells. TNBC: triple-negative breast cancer; H-Score: histological score; %SMA+T.Cells: percentage of positive cells. Note: *p < .05, **p < .01, ***p < .001, one-tailed.

		TNBC molecular type
%SMA+T.Cells	Pearson's r	0.386
	p-value	0.086
	95% CI upper	1.000
	95% CI lower	-0.088
	Spearman's rho	0.506*
	p-value	0.032
	Kendall's Tau B	0.399*
	p-value	0.031
H-Score	Pearson's r	0.458*
	p-value	0.050
	95% CI upper	1.000
	95% CI lower	-0.001
	Spearman's rho	0.506*
	p-value	0.032
	Kendall's Tau B	0.375*
	p-value	0.040

We summarized in Table 8 all significant correlations found for SMA expression in tumor cells, dependent on tumor score and clinicopathologic parameters.

**Table 8:** Conclusive table related to significance for each subgroup and clinicopathologic parameters. NS: not significant; TNBC: triple-negative breast cancer.

Parameters	Subgroup		
	αSMA_TS^low^	α​​​​​​​SMA_TS^med^	α​​​​​​​SMA_TS^high^
Tumor score	4	5-6	7-8
Menopausal status	NS	NS	p = 0.032
Molecular subtype	NS	NS	p = 0.050
Tumor size (T)	NS	p = 0.005 (TNBC-related)	p = -0.002
Nodal status (N)	NS	p = 0.048	NS
Proliferation rate (Ki67)	NS	NS	p= -0.039
Tertiary lymphoid structures (TLS)	NS	NS	NS
Immature blood vessels (IBV)	p = 0.029	NS	NS
Lymphovascular invasion (LVI)	NS	p < 0.001	p = -0.032
Perineural invasion (PNI)	NS	p = 0.007	p = -0.011
Recurrence	NS	p = 0.043 (partial)	p = -0.009
Survival	NS	p = -0.013	NS

## Discussion

αSMA (ACTA2) overexpression in breast cancer is extensively studied in relation to CAFs from tumor stroma [10, 11]. Data about αSMA expression in BC malignant cells are very limited, controversial, and mostly related to epithelial-mesenchymal transition [21]. Additionally, ACTA2 expression is especially associated with the BC basal phenotype [22]. This is also confirmed in the present study, where we reported a significant correlation between the TNBC phenotype, the percentage of αSMA-positive cells, and the H-score in the αSMA_TS^high^ subgroup but not in the αSMA_TS^low^ or αSMA_TS^med^ subgroups. Peng et al. [20] experimentally demonstrated that ACTA2-AS1 serves as a tumor suppressor and dramatically inhibits tumor cell proliferation, invasion ability, and progression of MDA-MB-231 breast cancer cells. We validated these experimental results here on human BC tissue specimens for the αSMA_TS^high^ subgroup. A high αSMA expression in tumor cells from our cases inhibited proliferation, LVI, PNI, and recurrence, with this finding being supported by inverse correlations obtained for all these parameters in the αSMA_TS^high^ subgroup. This is the first report on αSMA impact on clinicopathological parameters in human BC. Most papers published previously used BC cell lines, where the interrelation with clinicopathologic parameters was limited. Scattered data mentioned mutual interactions between αSMA tumor expression and other factors such as TP53 mutation or estrogen receptor (ER)/progesterone receptor (PR) status. Kim et al. demonstrated that TP53 activation induced αSMA tumor expression in tamoxifen-resistant cancer cells [16]. The same authors observed that cases with αSMA expression in ER-positive cancer cells had a poor prognosis [16]. This aligns with our findings for the αSMA_TS^med^ subgroup. In this group, more than 60% of cases showed ER positivity. This subgroup was the only one in this study where αSMA overexpression had a significant negative impact on survival, most likely due to ER presence in cancer cells.

Recently, Yadav et al. [18] identified several subgroups of TNBC cancer and a poor-prognosis Luminal A subgroup based on αSMA overexpression using single-cell multiplex imaging. We also found that in the αSMA_TS^low^ subgroup, scored as 4, there were no TNBC or Luminal A cases, while in the αSMA_TS^med^ and αSMA_TS^high^ subgroups, Luminal and TNBC subgroups predominated.

Apparently controversial but interesting data were found here regarding the correlation between αSMA tumor expression and invasion (lymphovascular-LVI, perineural-PNI) and recurrence. While in the αSMA_TS^med^ subgroup, actin-expressing tumor cells seem to favor invasion (both LVI and PNI) and recurrence, in the αSMA_TS^high^ subgroup, a high content of αSMA in tumor areas induced an inverse correlation with invasion and recurrence. This divergent data seems to be based on different pathogenic mechanisms involving αSMA-positive cancer cells. For the αSMA_TS^med^ subgroup, actin expression in tumor cells was strongly positively correlated with LVI, PNI, and R. This may be explained by the high hormone receptor positivity in this subgroup, which in turn may favor invasion through the activation of BC circulating tumor cells (CTCs), as previously described in several papers [23-25]. The actin cytoskeleton is highly dynamic, and an increase in αSMA expression may induce high BC cell rigidity, as reported in previous studies [26], which in turn could be negatively correlated with cell motility and subsequently with LVI and PNI. Last but not least, αSMA expression in the tumor microenvironment modulates malignant cell invasion. In one of our previous studies, we reported that αSMA expression in CAFs from tumor stroma strongly influences PNI and LVI differently, depending on BC molecular subtypes. Tumor stromal microenvironment stiffness strongly influences BC cell invasion ability [27-30].

Most of the cases (statistically significant) from the αSMA_TS^high^ subgroup are women in menopausal status, where the hormonal microenvironment is completely altered. An increase in tumor cell αSMA expression in the αSMA_TS^high^ subgroup may be induced by the lack of hormonal stimulation during menopause, which in turn reduces tumor cell invasion and subsequently recurrence. No data are currently available in the literature regarding this subject, so our hypothesis may be strengthened by further studies focused on this issue. We will continue to investigate this aspect in a larger number of cases and for each molecular subtype.

No data on the impact of menopausal status on αSMA expression in tumor cells have been previously reported. We found that most cases scored as 7 and 8 for αSMA expression in tumor cells were diagnosed in menopausal patients. This may be explained by the altered hormonal microenvironment during menopause.

## Conclusions

αSMA expression in tumor cells is highly heterogeneous and dependent on several factors. Its expression in tumor cells specifically impacts invasion, recurrence, and survival through direct interaction with the tumor microenvironment. Based on digital image analysis of αSMA expression in tumor cells, we defined three main subgroups of BC with different behavior, recurrence, and survival. Further studies must be developed to elucidate the impact of tumor cell αSMA expression on other clinicobiological parameters, such as therapy response, disease-free survival, or recurrence.
